# Effects of Extract of *Arrabidaea chica* Verlot on an Experimental Model of Osteoarthritis

**DOI:** 10.3390/ijms20194717

**Published:** 2019-09-23

**Authors:** Cleydlenne Costa Vasconcelos, Alberto Jorge Oliveira Lopes, Emerson Lucas Frazão Sousa, Darleno Sousa Camelo, Fernando César Vilhena Moreira Lima, Cláudia Quintino da Rocha, Gyl Eanes Barros Silva, João Batista Santos Garcia, Maria do Socorro de Sousa Cartágenes

**Affiliations:** 1 Biological and Health Sciences Center, Federal University of Maranhão, Av. dos Portugueses 1966, São Luís, MA 65085-580, Brazil; lopesajo@gmail.com (A.J.O.L.); e.lucasfrazao@gmail.com (E.L.F.S.); leno.camelo@gmail.com (D.S.C.); fernandovilhena15@gmail.com (F.C.V.M.L.); gyleanes@fmrp.usp.br (G.E.B.S.); jbgarcia@uol.com.br (J.B.S.G.); 2 Department of Chemistry, Federal University of Maranhão, São Luís, MA 65085-580, Brazil; claudiarocha3@yahoo.com.br

**Keywords:** pain, inflammation, phytotherapy, antinociception, pariri, bioinformatics, molecular docking, plants

## Abstract

The aim of this study was to analyze the analgesic potential of *Arrabidaea chica* extract (EHA) as an alternative to osteoarthritis (OA) treatment. Thus, the extract was initially evaluated by the cyclooxygenase inhibition test. The analgesic effect of the extract, in vivo, was also verified in a model of OA induced by sodium monoiodoacetate (2 mg). EHA was administered to rats at doses of 50, 150, and 450 mg/kg between 3 and 25 days after OA induction. The animals were clinically evaluated every 7 days, euthanized at 29 days, and the liver, spleen, kidney and knee collected for histopathological analysis. The chemical composition of EHA was identified by HPLC-MS and the identified compounds submitted to molecular docking study. The results showed that the extract promoted cyclooxygenase inhibition and produced significant improvements in disability, motor activity, hyperalgesia, and OA-induced allodynia parameters, in addition to improvements in the radiological condition of the knees (but not observed in the histopathological study). Chemically the extract is rich in flavonoids. Among them, we evidence that amentoflavone showed very favorable interactions with the enzyme COX-2 in the in silico analysis. Thus, it is concluded that *A. chica* has important analgesic properties for the treatment of OA.

## 1. Introduction

The plants have contributed significantly to the development of new therapeutic strategies for the treatment of several diseases, mainly through the secondary metabolites, that can interfere in the performance of mediators of the inflammatory process, second messenger production, and expression of transcription factors [1,2,3]. The *Arrabidaea chica* (Humb. And Bonpl.) B.Verlot (Bignoniaceae) plant species used in this study has been distinguished by its pharmacological potential, associated mainly to its antioxidant, astringent, antimicrobial, antitumor, and anti-inflammatory activities [4,5].

Osteoarthritis (OA) is the disease in which the inflammatory process plays a critical role in its pathogenesis [6]. Thus, *A. chica* could represent a potential alternative to treatment. OA is characterized by progressive destruction of articular cartilage, synovial inflammation, changes in the subchondral bone and peri-articular muscle, pain and has a generally slow progression [7,8]. As for the inflammatory process, increased levels of pro-inflammatory cytokines such as interleukin 1β (IL-1β) and tumor necrosis factor alpha (TNF-α) decrease collagen synthesis and increase catabolic mediators such as metalloproteinases and other substances inflammatory [8,9]. As for existing pharmacological interventions for OA pain, these are often insufficient or poorly tolerated, and OA pain generally remains refractory to available treatments [10,11]. In addition to adverse drug effects resulting from long-term use, non-steroidal anti-inflammatory drugs (NSAIDs) provide temporary relief but can cause gastrointestinal problems, renal dysfunction and elevated blood pressure, among others [12,13].

OA is also an important public health issue related to aging, which affects about 40% of adults over 70 years of age and generates great socioeconomic impact since it can lead to functional disability of the individuals, affecting the quality of life in addition to producing high costs for health services [14,15,16]. Thus, the present study aimed to investigate the potential of *A. chica* extract as an alternative to the treatment of OA.

## 2. Results

### 2.1. Inhibition of Cyclooxygenase 1 and 2

**Figure 1:**
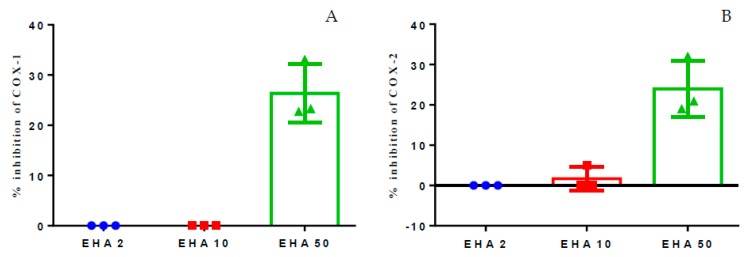
Percent inhibition of in vitro COX-1 (**A**) and COX-2 (**B**), induced by the hydroethanolic extract of *A. chica,* tested in three concentrations: 2, 10, and 50 μg/mL.

In vitro inhibition assays of cyclooxygenase-1 and 2 (COX-1 and COX-2) were performed in order to investigate whether the hydroethanolic extract of *A. chica* has inhibitory effects on these enzymes. The results of the assay showed that the extract has an inhibitory effect, inhibiting up to 30% of COX-1 and COX-2 in the highest concentration tested, 50 μg/mL. However, concentrations of 2 and 10 μg/mL failed to produce any inhibitory effect on the enzymes, or the effect was very low. When comparing the effects of the extract on COX-1 and COX-2, it was possible to verify that the extract does not appear to be selective for COX-1 or COX-2, since at 50 μg/mL the extract inhibited around 20 to 30% in both the isoforms (Figure 1A,B).

### 2.2. Clinical Evaluations

#### 2.2.1. Evaluation of Motor Activity/Forced Deambulation (Rotarod Test)

**Figure 2:**
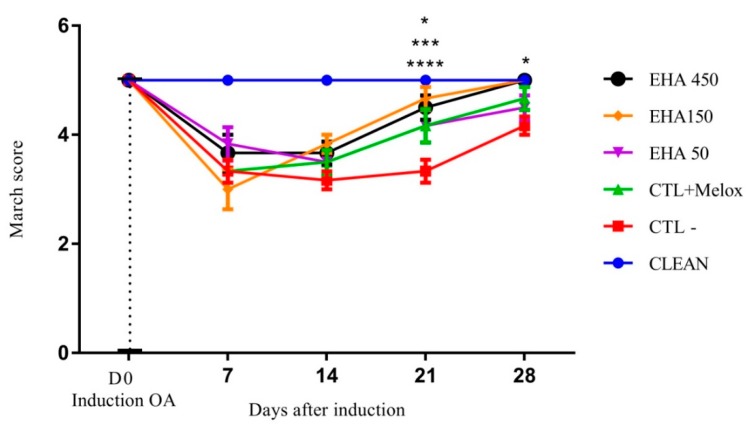
Effects of the *A. chica* extract on the motor activity/march score of rats with induced osteoarthritis (OA). The induction of OA was done by the injection of MIA (2 mg) in the knee of Wistar rats. After 3 days, the animals received oral saline (CTL−), Meloxicam (CTL + Melox), and *A. chica* extract at doses 50, 150, and 450 mg/kg (EHA50, EHA150, and EHA450). After 7, 14, 21, and 28 days of induction, the motor activity of the animals was measured to evaluate the rotarod score. The data are represented in mean ± standard deviation of the means. The healthy group is represented by animals without osteoarthritis and without treatment (CLEAN). * represents significant differences, with *p* < 0.05; *** with *p* < 0.0005; **** with *p* < 0.0001 comparing the treatments to the saline group.

The evaluation of the motor activity initially showed that the induction of OA was effective, since the first evaluation after induction (D7) showed a significant difference between the induced groups (EHA50, EHA150, EHA450, CTL + Melox, CTL−, and healthy control), marked by decreased motor activity of animals with induced OA. This decrease was also observed on day 14, demonstrating that, until this period, the treatments did not produce any significant improvement in motor activity (Figure 2). On day 21 after the induction of OA, all treated groups, namely, EHA50 (*p* = 0.026), EHA150 (*p* < 0.0001) EHA450 (*p* = 0.0004), and CTL + Melox (*p* = 0.026) showed a significant difference in relation to the saline group, demonstrating that the extract at this concentration is able to promote improvement in motor activity. On day 28, these significant differences in relation to the saline group were also observed in relation to the extract EHA450 (*p* = 0.028) and EHA150 (*p* = 0.028) (Figure 2).

#### 2.2.2. Incapacitation/Weight Distribution Test on Hind Legs (Weight Bearing)

**Figure 3:**
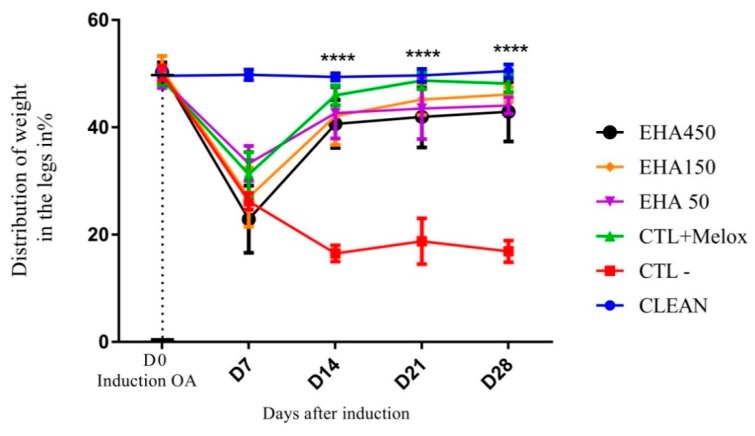
Effects of the extract *A. chica* on the degree of incapacitation of rats with induced osteoarthritis. The induction of osteoarthritis was done by the injection of MIA (2 mg) in the knee of Wistar rats. After 3 days, the animals received oral saline (CTL−), Meloxicam (CTL + Melox) and extract of *A. chica* at doses 50, 150, and 450 mg/kg (EHA50, EHA150, and EHA450). After 7, 14, 21, and 28 days of induction, the weight distribution on the hind legs of the animals was measured for the assessment of the incapacity by means of the Weight Bearing test. The data are represented in mean ± standard deviation of the means. The healthy group is represented by animals without osteoarthritis and no treatment (CLEAN). **** represents significant differences, **** with *p* < 0.0001 comparing the treatments to the saline group.

The analysis of the data related to joint incapacitation, evaluated using the percentage of weight distribution in the legs, showed that the induction of OA was effective, since on the seventh day after induction we observed a significant difference between the induced OA group and the control group (CLEAN). On days 14, 21, and 28 post-induction, all treated groups (EHA50, EHA150, EHA450, CTL + Melox) showed significant improvement in disability, and differed significantly from the saline group (CTL−) (*p* < 0.0001) (Figure 3).

#### 2.2.3. Mechanical Hyperalgesia (Randall Selitto Test)

**Figure 4:**
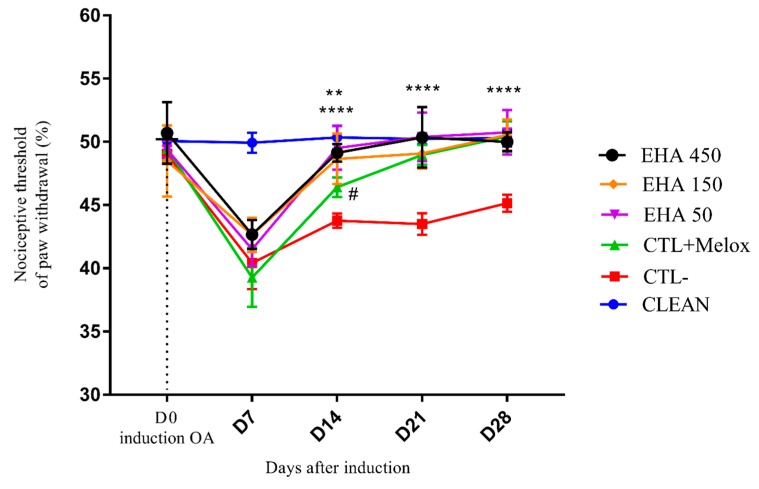
Effects of *A. chica* extract on mechanical hyperalgesia in rats with induced osteoarthritis (OA). The induction of OA was done by the injection of MIA (2 mg) in the knee of Wistar rats. After 3 days, the animals received oral saline (CTL−), Meloxicam (CTL + Melox) and extract of *A. chica* at doses 50, 150, and 450 mg/kg (EHA50, EHA150, and EHA450). After 7, 14, 21, and 28 days of OA induction, the nociceptive threshold of withdrawal of the affected paw was measured by indirect evaluation with Randall Sellito apparatus. The data are represented in mean ± standard deviation of the means. The healthy group is represented by animals without osteoarthritis and without treatment (CLEAN). ** represents significant differences, with *p* < 0.005; **** with *p* < 0.0001 comparing the treatments to the saline group. # represents significant differences, with *p* < 0.05 comparing the EHA450 with CTL + Melox.

The animals, when evaluated, for mechanical hyperalgesia showed a decrease in the nociceptive paw withdrawal threshold on day 7 after MIA injection. This decrease occurred in all induced groups (EHA50, EHA150, EHA450, CTL + Melox, and CTL−), as expected, confirming the success of OA induction. On day 14 of the experiment, all treated groups EHA450, EHA150, EHA50 (*p* < 0.0001), and CTL + Melox (*p* = 0.003) showed nociceptive threshold increase, with significant improvement in mechanical hyperalgesia when compared to the saline group; the EHA450 extract being statistically better (*p* = 0.02) until drug meloxicam. Also, observations on day 21 and 28 of the analysis, showed that the EHA450, EHA150, EHA50 and CTL + Melox (*p* < 0.0001) treated groups continued to differ significantly from the saline group (Figure 4). Thus, the extract was able to induce significant improvements in hyperalgesia by induced OA from day 14 of the analysis, demonstrating its nociceptive potential.

#### 2.2.4. Mechanical Allodynia (von Frey Test)

**Figure 5:**
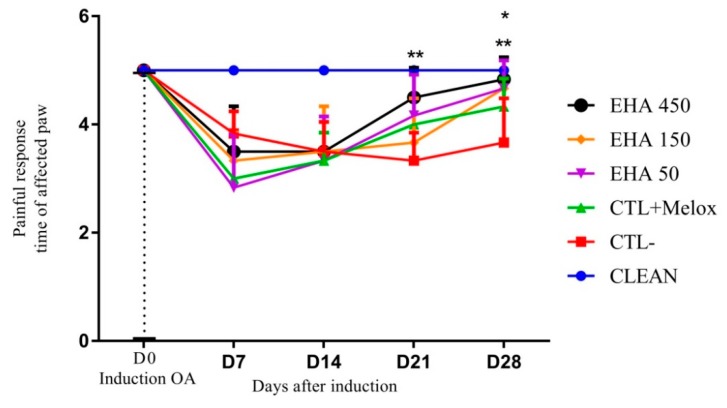
Effects of *A. chica* extract on mechanical allodynia in rats with induced osteoarthritis (OA). The induction of OA was made by the injection of MIA (2 mg) in the right knee of Wistar rats. After 3 days, the animals received oral saline (CTL−), Meloxicam (CTL + Melox) and *A. chica* extract at doses 50, 150, and 450 mg/kg (EHA50, EHA150, and EHA450). After 7, 14, 21, and 28 days of OA induction, mechanical allodynia was measured by indirect evaluation with the von Frey test. The data are represented in mean ± standard deviation of the means. The healthy group is represented by animals without osteoarthritis and without treatment (CLEAN). * represents significant differences, with *p* < 0.05; ** with *p* < 0.005 comparing the treatments to the saline group.

The results of mechanical allodynia evaluation first demonstrated the efficacy of fear of induction by MIA, since the animals immediately after induction (D7) had a significant reduction of the response time to the stimulus compared to the group that was not induced (CLEAN). On day 21 after induction the group treated with extract at the concentration of 450 mg/kg (EHA450) showed a significant difference (*p* = 0.0015) in relation to the saline group, demonstrating that the extract at this concentration is able to promote improvement in allodynia mechanics. On day 28 this result was also observed, but in addition to the EHA450 extracts, the extracts of lower concentration (EHA50) and (EHA150) also produced significant improvement (*p* = 0.0108) in allodynia compared to the saline group (Figure 5).

### 2.3. Radiographic Analysis

**Figure 6:**
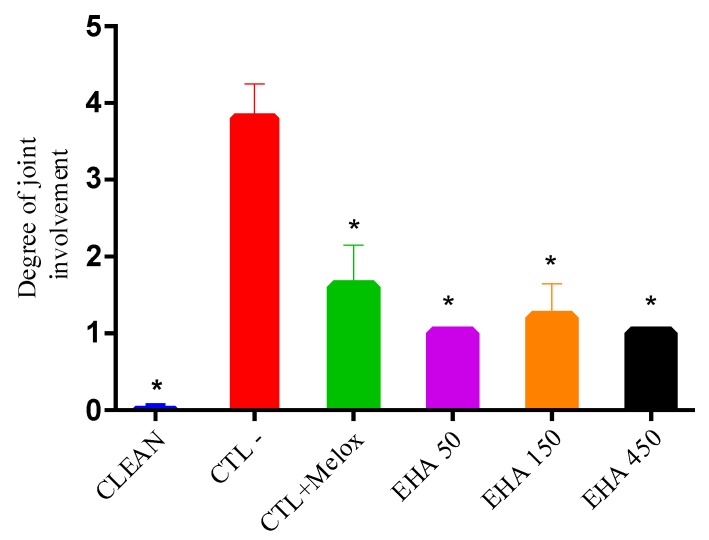
Degree of joint involvement in rats treated with *A. chica* extract (EHA50, 150, and 450 mg/kg, orally), Meloxicam (CTL + Melox) and saline (CTL–) administered from day 3 after OA induction. This analysis was performed with the knees collected on day 29 after OA induction. The data are represented according to classification proposed by Kellgren–Lawrence. * represents significant differences, with *p* < 0.05, between the groups treated with *A. chica* extract and the saline group (CTL−).

**Figure 7:**
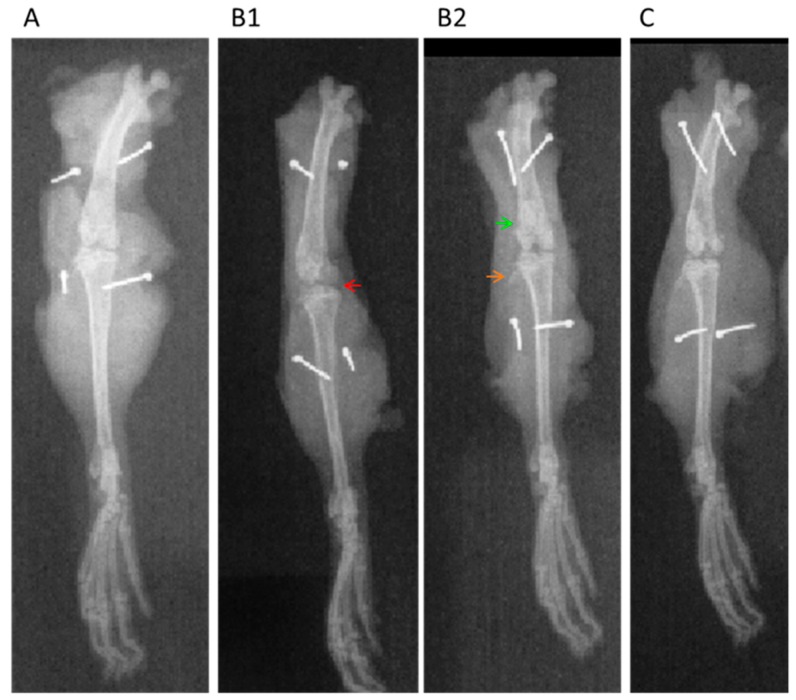
Knee radiographs in the antero-posterior projection. **A**-RX from the healthy rat paw (CLEAN); **B1** and **B2**-RX from the rat paw with experimental model of MIA-induced osteoarthritis–saline group; **C**-RX, *A. chica* extract 450 mg/kg treated group. This analysis was performed with the knees collected on day 29 after OA induction. Arrowhead: red—indicating a remarkable narrowing of the joint space and deformity in the bone contour; green—subchondral sclerosis; orange—large osteophytes.

In the radiographic analyzes, it was observed that in the group without osteoarthritis (CLEAN), the scores were minimal (grade 0; Figure 6), with preservation of the joint space, without subchondral sclerosis and no formation of osteophytes (Figure 7A) which would be the main features of OA. In the group that had OA induction by MIA and received no treatment (CTL−), a high score was observed, reaching grade 4 (Figure 6), with common radiographic characteristics of the disease, such as reduction of articular space, marked subchondral bone sclerosis, and intense formation of osteophytes as seen in Figure 7(B1,B2). In the groups treated with extract (EHA50, EHA150, and EHA450 (Figure 7C) and Meloxicam, these common radiographic features of OA were much less evident and the score did not exceed grade 2 according to the classification of Kellgren–Lawrence (Figure 6). Thus, the extract in the three concentrations tested was able to significantly reduce (*p* < 0.05) the degree of joint involvement in relation to the saline group (CTL−) (Figure 6).

### 2.4. Histopathological Analysis

**Figure 8:**
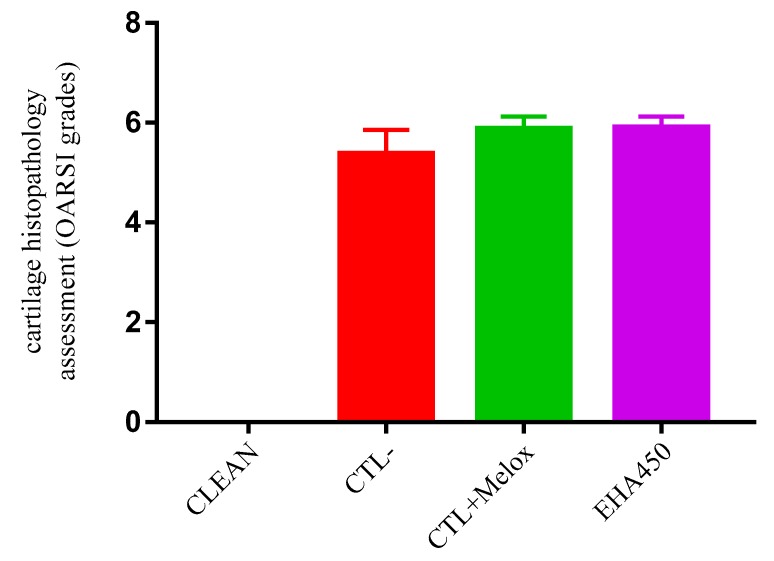
Histopathological evaluation of cartilage classified using the Osteoarthritis Research Society International (OARSI) scoring system. This analysis was performed with the knees collected on day 29 after OA induction. Groups: EHA450 = with sodium monoiodoacetate-induced osteoarthritis (MIA-induced OA) 2 mg and treated with *A. chica* hydroalcoholic extract 450 mg/kg; CTL− = with MIA-induced OA (2 mg) and treated with 0.9% sodium chloride saline; CTL + Melox = with MIA-induced OA (2 mg) and treated with meloxicam 0.5 mg/kg; CLEAN = untreated and uninduced OA, healthy. *Y*-axis: Histopathological evaluation of cartilage (OARSI histological classification: Grade 0—surface intact and cartilage intact; Grade 1—surface intact; Grade 2—surface discontinuity; Grade 3—vertical fissures; Grade 4—erosion; Grade 5—denudation; and Grade 6—deformation).

**Figure 9:**
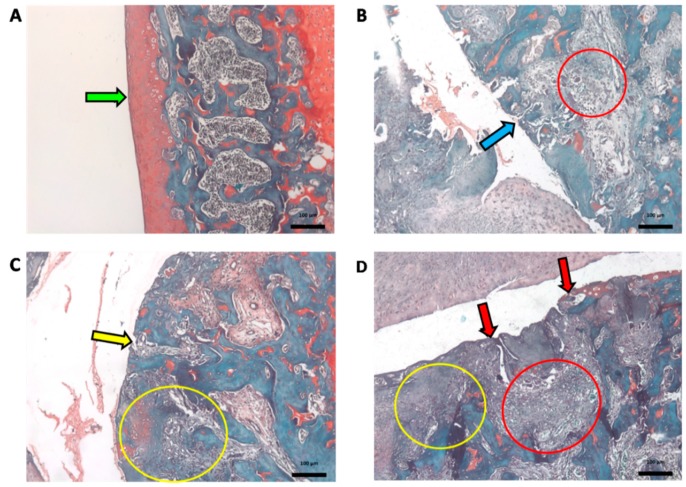
Sections of articular cartilage with different grades of degenerative changes in osteoarthritis (OA) classified using the Osteoarthritis Research Society International (OARSI) scoring system.This analysis was performed with the knees collected on day 29 after OA induction. (**A**) Example of CLEAN group, untreated and uninduced OA, healthy (Grade 0) normal cartilage. (**B**) Example of CTL-group treated with 0.9% saline. (**C**) Example of EHA450 group treated with *A. chica* hydroalcoholic extract 450 mg/kg. (**D**) Example of CTL + Melox group. B, C, and D with degrees on average between 5.4 and 5.9. Green arrow indicates normal cartilage lining (intact surface and cartilage); blue arrow indicates microfractures; red arrow indicates erosion with bone expositon; red circles indicate repair fabric; yellow arrowindicates complete erosion; yellow circles indicate fibrocartilage formation and bone remodeling. (O-safranin stain; Bar = 100 µm; 200×).

Histopathological evaluation using the OARSI scoring system (see Materials and Methods item 4.5) revealed that the treatment with *A. chica* extract at the highest dose tested (EHA450) was not able to inhibit the OA-induced joint compromise. The OARSI classification system group EHA450 obtained an average grade rating of 5.9 (±0.22), similar to the saline group (CTL−) of 5.4 (±0.47) and the meloxicam-treated group (CTL + Melox) of 5.9 (±0.25) (Figure 8). These grades above 5, according to the OARSI classification, indicate greater articular cartilage injury in these animals, recognized by denudation, complete erosion of the hyaline cartilage to the level of mineralized cartilage and/or bone, microfractures, as well as fibrocartilaginous bone surface repair (Figure 9).

**Figure 10:**
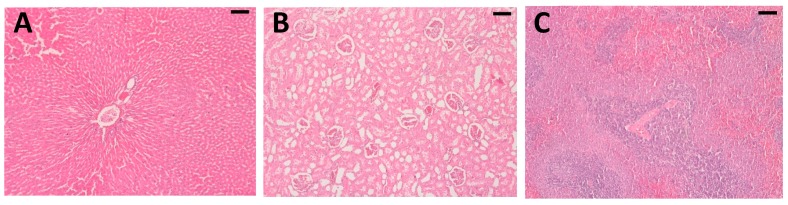
Exemplary histological sections of the liver (**A**), kidneys (**B**), and spleen (**C**) of EHA450 group. The images show no signs of ischemia, necrosis, cell injury and/or inflammation. (Hematoxylin-Eosin—HE stain, Bar = 100 µm, 100×).

Histopathological analyses of the liver, spleen and kidney showed no pathological changes, even at the highest administered dose of *A. chica* extract (450 mg/kg) (Figure 10), thus demonstrating that the extract did not induce any toxicity.

### 2.5. Chemical Analysis

**Figure 11:**
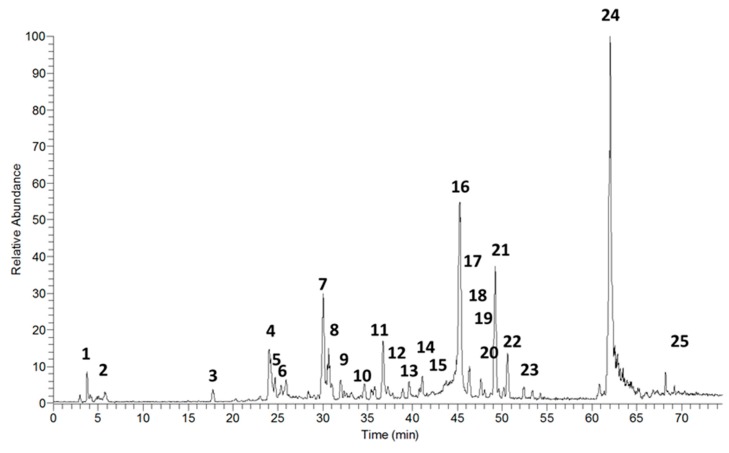
Chromatogram of total ions obtained by HPLC-PDA-ESI-IT-MS/MS, positive mode, from the hydroethanolic extract of *A. chica* leaves.

The HPLC-PDA-ESI-IT-MS/MS analysis, in the positive mode, showed the presence of 25 compounds in the hydroethanolic extract of *A. chica* leaves (Figure 11); among them, 22 were identified by dereplication, comparing the masses, retention times, and fragments obtained in mass spectrometry, with the literature (Table 1).

### 2.6. In silico Analysis

To our molecular docking analysis, we used all metabolites identified by HPLC-MS on *A. chica* extract. On general, all metabolites showed satisfactory parameters affinity with the COX-2 structure. In addition to these molecules, molecular docking of the commercial non-steroidal anti-inflammatory drug meloxicam was performed, with affinity parameters of −8.82 kcal·mol^−1^ and 0.34 µM from free binding energy and the inhibition constant, respectively, while among the *A. chica* metabolites, the best affinity parameters were shown to be amentoflavone and quercetin-o-gallate with −9.21 and −8.86 kcal·mol^−1^ from free binding energy, respectively, and 0.11 and 0.32 µM from the inhibition constant, respectively; these parameters being superior to those presented by meloxicam. The results of the binding energy values of all compounds are shown in Table 2.

**Figure 12:**
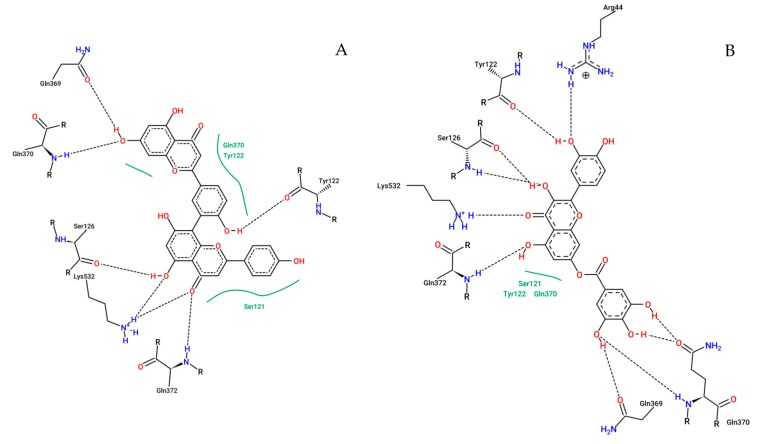
2D representation of the interactions of COX-2 residues with amentoflavone (**A**) and quercetin-o-gallate (**B**). Dashed lines represent hydrogen bonds; green lines represent van der Waals interactions.

The map from interactions of the amentoflavone and quercetin-o-gallate with COX-2 amino acids identified in selected configurations obtained through molecular docking calculations are displayed in Figure 12.

## 3. Discussion

Herbal medicines have become considerably popular to relieve the symptoms of various diseases, including OA [34]. However, available scientific data are still insufficient to support the use of these products in the clinical management of OA. However, the growth of studies in this sense brings expectations regarding the arrival of reliable, efficient, and safe herbal products that meet the criteria of modern medicine [35].

In order to meet these expectations, the present study evaluated pain behavior in an experimental OA model using *A. chica* extract as an alternative to traditional pharmacological treatments. Since many studies have proven its therapeutic potential, including its anti-inflammatory activity [36,37,38], and due to the fact that OA is a disease in which the inflammatory process plays a central role in the pathogenesis [8], we evaluated this plant against OA.

The present study shows the anti-inflammatory and analgesic properties of *A. chica* extract through in combo studies: In vitro, by inhibition of cyclooxygenase; in vivo, due to the improvement in clinical and radiological parameters of animals with MIA-induced OA; and also by the demonstration of potential mechanisms of action of *A. chica* secondary metabolites through in silico studies. Chemical analysis of the extract allowed the identification of a large number of molecules with bioactive potential, including metabolitoes were described for the first time in the species.

In this context, *A. chica* extract was evaluated for its ability to inhibit COX enzymes in vitro. COX-2 participates in inflammatory and painful processes, being responsible for the metabolization of arachidonic acid resulting from the action of phospholipase A_2_ on membrane lipids, which produce intermediates, prostaglandin G_2_, and prostaglandin H_2_ which are isomerized to prostanoids such as prostaglandins, prostacyclins, and thromboxanes [39]. Due to the role this enzyme plays in inflammatory processes, it is the target of non-steroidal anti-inflammatory drugs and have represented a potential target for action of new compounds. And in this study, *A. chica* extract was able to inhibit COX-2 and COX-1 by up to 30% (Figure 1A,B), demonstrating its anti-inflammatory potential.

The antinociceptive activity of *A. chica* extract was observed from the improvement of motor activity from day 21 after OA induction, in all doses, similar to meloxicam (Figure 2). Treatment with the dose of 500 mg/kg of *Spinacia oleracea* L. extract also showed improvement in the motor activity of the animals, but only later (day 28) [40]. 

Treatment with *A. chica* extract also had antinociceptive effects, significantly reducing the weight distribution deficit between the left and right paws, improving OA-induced disability from 14 days after induction, in all doses (Figure 3). The paw weight distribution test is an indicator of OA progression and reveals the efficacy of anti-inflammatory compounds, as observed in the study with an isolated compound from *Zingiber zerumbet* (L.) Smith. which was able to reduce joint discomfort in this same model [41].

The extract also improved the hyperalgesia threshold from day 14 onwards after OA induction, also in all doses (Figure 4). Hyperalgesia is characterized by a painful response accentuated by a previously painful stimulus [42]. Treatment with *Entada pursaetha* DC., hydroethanolic extract 30, 100, and 300 mg/kg also significantly reduced hyperalgesia in the MIA-induced OA model on days 7, 14, and 21. The authors suggest that this effect may be linked to the reduced production of inflammatory mediators responsible for peripheral and central sensitization of pain and hyperalgesia in the model in question [43].

The results of the clinical evaluations of the present study also show that *A. chica* extract 450 mg/kg, from day 21 of treatment, significantly reduced allodynia (pain associated with a stimulus that would normally not cause pain), and for all doses on day 28 [42] (Figure 5). Lima [38] also observed this analgesic effect of *A. chica* extract in an experimental model of neuropathic pain induced by sciatic nerve compression, hence corroborating the results found here. Similar results were also found by our group in Calado et al. [44] when they analyzed the effects of the hydroethanolic extract of *Chenopodium ambrosioides* L. leaves in an experimental OA model using the same methodology.

A possible explanation for improvement of these clinical aspects could be the anti-inflammatory activity of the flavonoids present in the extract, which may be able to inhibit COX, and produce this action, as reported by [45]. *A. chica* extract also demonstrated this effect by inhibiting nuclear transcription factor kappaβ, which consequently prevents the formation of inflammatory mediators such as iNOS, COX-2, 5-LOX, and phospholipase A_2_ [18]. Studies by Lima et al. [32] also observed that in lipopolysaccharide-induced peritonitis in mice, oral pretreatment with hydroethanolic extract of the leaves or with the isolated compound 4′,6,7-trihydroxy-5-methoxyflavone (5-o-methyl scutellarein) led to decreased leukocyte migration to the peritoneal cavity, as well as a reduction in proinflammatory cytokine concentrations (TNFα and IL-1β).

Regarding the radiographic findings of the present study, they showed that after OA induction, subchondral sclerosis, osteophytes, and decreased joint space were found in the evaluated knees. Although *A. chica* extract was unable to prevent the most frequent radiological changes that occur in OA, the animals treated with herbal medicine had lower degrees of joint changes according to the Kellgren–Lawrence classification. However, histopathological analyses did not show this improvement for the *A. chica*-treated EHA group.

Importantly, radiological parameters are not always associated with clinical or histological results. This factor generates difficulties in the interpretation of the pain phenomenon in OA; however, people with radiographic alterations compatible with OA have a higher chance of presenting pain than individuals without this type of alteration [46,47].

In the chemical analysis of the extract, 22 compounds were identified, most of which belong to the group of flavonoids (Table 1) that comprise an important class of natural pigments, having a chemical structure consisting of two aromatic rings linked by a chain of three atoms forming an oxygenated heterocycle. The degree of oxidation and the substitution pattern of ring C rank the flavonoids and the substitution pattern on rings A and B specifically defines each compound [48].

Flavonoids in general have been attributed important biological activities, among them an important anti-inflammatory action, due to the ability of these compounds to modulate the action of cellular components involved in the mechanism of inflammation such as pro-inflammatory cytokines TNF-α and IL-1, and the activity of arachidonic acid pathway enzymes such as cyclooxygenase and lipoxygenase [47,48]. Results such as these were observed by studying extract and fractions of *Tabernaemontana catharinensis* DC. leaves, which provide the ability to inhibit leukocyte migration and significantly decreased the levels of various proteins such as MPO, interleukin (IL)-1β, and tumor necrosis factor TNF-α [49]. These actions are attributed at least in part to the flavonoids present in the plant.

Among the 22 compounds identified, it should be noted that 12 are being reported for the first time in species *A. chica*. They are: 2′-hydroxy-a-naphthoflavone; 5,7-dimethoxy-4′-hydroxyflavone; quercetin-o-gallate; quercetin-o-glucoside; amentoflavone; isorhamnetin-3-o-glucoside; chrysoeriol; chrysoeriol-o-glucoside; isorhamnetin; cirsimarin; hyperine 6″ gallate; and catechin dimer.

Considering the potential of these compounds as therapeutic agents and with the encouraging results of the in vitro and in vivo studies, the in silico study was carried out in order to verify the possible pathways of action of the metabolites identified in the extract. Thus, the metabolites had their interactions with the structure of the COX-2 enzyme evaluated through molecular doping. Molecular docking data show that amentoflavone and quercetin-o-gallate were among the metabolites which had more favorable interactions with COX-2. 

Negative binding-free energy values between the binder and the macromolecule indicate favorable interactions [50]. The active site of COX-2 involves residues Arg120, Tyr355, Tyr385, Glu524, and Ser530 where arachidonic acid binds, thus forming prostaglandins [51,52]. The result of molecular docking demonstrated that amentoflavone and quercetin-o-gallate performed a large number of interactions (hydrogen bonds and van der Walls interactions) with residues neighboring this region (Figure 12). Silva and colleagues [53] have identified through molecular docking and molecular dynamics simulations that the ursolic acid inside all metabolites identified on the ethylacetate fraction of *Borreria verticillata* (L.) G. Mey. was responsible for the anti-inflammatory activity of this plant species. The ursolic acid was selected by in silico assay and when evaluated isolated in vivo on mice, showed anti-inflammatory activity higher than indomethacin.

Amentoflavone showed anti-inflammatory activity besides suppressing the production of nitric oxide (NO) and prostaglandin E_2_ (PGE_2_) in RAW264.7 cells [54]. Also, amentoflavone promotes the downregulation of COX-2 and iNOS levels in cancer cells. This activity is also well associated with the suppression of PGE_2_ biosynthesis [55]. Evaluated against an ulcerative colitis model on mice, the amentoflavone showed decreases in the mucosal injury by lowered colonic wet weight as well as vascular permeability and diminished lactate dehydrogenase (LDH) and myeloperoxidase (MPO) activity reflecting reduced leukocyte infiltration and reducing significantly the tumor necrosis factor-alpha (TNF-α), interleukin-1 beta (IL-1β), and IL-6 levels as well as the expression of iNOS and COX-2 [56].

## 4. Materials and Methods

### 4.1. Collection and Processing of Plant Species

The leaves of *Arrabidaea chica* were collected in the urban area of São Luis city, Maranhão state, Brazil, in May 2016 (2° 33′29″ S, 44° 18 ′30″ W) between 5 to 6 p.m. A sample of the plant was deposited in the “Atico Seabra” Herbarium, Federal University of Maranhão (UFMA), and was identified and cataloged in register 1.067. For preparation of the extract, the collected plant material was dried at 40 °C in an air circulating oven and then pulverized in an electric mill to obtain the powder, which was impregnated in 70% ethanol in a ratio of 1:4 (*m*/*v*) and placed in maceration, under daily manual agitation. The alcoholic extraction of the macerate was carried out by three successive changes every 72 h, with the renovation of the solvent. At the end of this process, the extracts were pooled and gauze filtered. The filtrate was concentrated in a rotary evaporator under reduced pressure and at a temperature of 40 °C. From this process, the hydroethanolic extract (EHA) was obtained.

### 4.2. In vitro Activity on Cyclooxygenase

Cyclooxygenase (COX) has been associated with the target of non-steroidal anti-inflammatory drugs and has been a potential target for the study of new drugs. Thus, in vitro tests of inhibition of COX by the hydroethanolic extract of *A. chica* were carried out, according to the instructions of the manufacturer of the enzymatic kit (Colorimetric COX Inhibitor Screening Assay Kit, Cayaman Chemical^®^, Ann Arbor, Michigan, USA.) and percent inhibition of COX-1 and COX-2, calculated from the means of the absorbance values, read at 590 nm. Inhibition assays were performed with extracts at three concentrations 2, 10, and 50 µg/mL in triplicate.

### 4.3. In vivo Experimental Studies

This study was conducted at the Experimental Laboratory for the Study of Pain (LEED). All procedures were approved in May 22, 2017 by the Ethics Committee on Animal Use of UFMA under No. 23115.000372/2017-09.

#### 4.3.1. Animals

Wistar rats (*Rattus norvegicus*) males, adults approximately 60 days old, which were procured from the Central Vivarium (Biotério Central) of Federal University of Maranhão (UFMA), São Luis, Brazil, were used in this study. These animals, throughout the experiment, were fed standard feed and water ad libitum and kept under a controlled temperature of 23 ± 1 °C and humidity of 40–60% under a 12 h light–dark cycle.

#### 4.3.2. Experimental Design

The animals were divided into 6 groups (n = 6, per group): Group 1—without OA and untreated (CLEAN); Group 2—with osteoarthritis and treated with saline (NaCl 0.9%, 0.1 mL/kg) (CTL−); Group 3—with osteoarthritis and treated with Meloxicam^®^ (0.5 mg/kg) (CTL + Melox) [57]; Groups 4, 5,and 6—with osteoarthritis and treated with *A. chica* hydroethanolic extracts at doses of 50, 150, and 450 mg/kg (EHA50, EHA150, EHA450), respectively.

**Figure 13:**
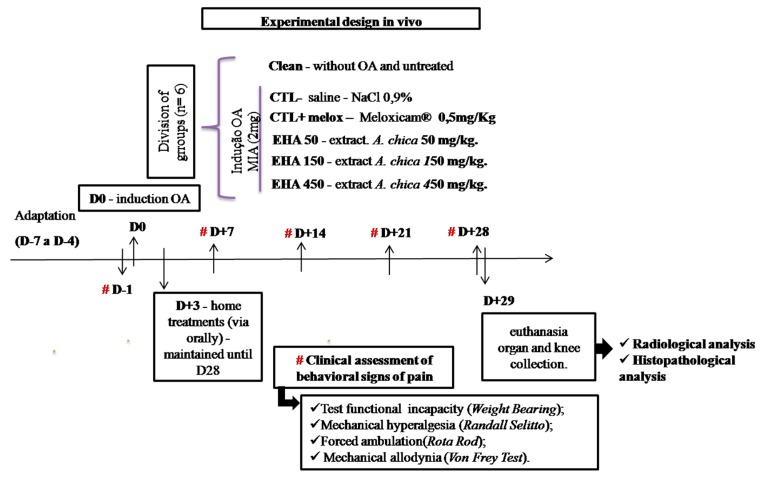
Experimental design.

The CLEAN group did not undergo any type of intervention. The other groups (CTL−, CTL + Melox, EHA50, EHA150, EHA450) received intra-articular injections of MIA (2 mg) for induction of osteoarthritis in the knee. And after three days, these groups received their respective treatments, as described above, orally (once daily) for 25 days (Figure 13).

These groups were evaluated clinically for disability, allodynia, mechanical hyperalgesia, and motor activity every 7 days, and were slaughtered on day 29 after initiation of OA induction. The animals were euthanized with a 2:1 anesthetic solution of ketamine hydrochloride (100 mg/kg) and xylazine hydrochloride (80 mg/kg), after which the blood of all animals was collected through the abdominal artery. Liver, spleen, and kidney were collected for histopathological analysis, as well as the right paw induced for radiographic and histopathological analyses (Figure 13).

#### 4.3.3. MIA-Induced OA Model

The animals were anesthetized by inhalation of isoflurane 1%. After certifying the anesthetic plane, a trichotomy was performed in the right knee and, subsequently, a topical solution of 10% iodopovidone was applied for local asepsis. An articular lesion was induced by a single intra-articular injection of 2 mg sodium MIA (diluted in a maximum volume of 25 μL) into the right knee through the patellar ligament [58,59].

#### 4.3.4. Clinical Evaluations

##### Evaluation of Motor Activity/Forced Deambulation (Rotarod Test)

The animals were placed on a swivel bar (IITC Life Science, Woodland Hills, CA, USA.) at a speed of 16 rpm for a period of 300 s. The use of the affected limb was assessed by forced deambulation. The use of the paw was graded using a numerical scale ranging from 5 to 1, where: 5 = normal use of the paw, 4 = mild claudication, 3 = severe claudication, 2 = intermittent disuse of affected paw, and 1 = complete disuse of affected paw [60].

##### Incapacitation/Weight Distribution Test on Hind Legs (Weight Bearing)

The animals were placed in a glass bowl angled and positioned so that each hind leg laid on different platforms. The weight exerted on each back paw (measured in grams) was evaluated for 5 s. The final measurement of weight distribution was the mean of three measurements [61]. Changes in the weight distribution on the paws were calculated as follows:

(1) $$\mathrm{Weight}\;\mathrm{distribution}\;(\%)=\frac{\mathrm{APW}}{\mathrm{APW}+\mathrm{CPW}}\times 100$$

where APW was affected paw weight and CPW was contralateral paw weight.

##### Mechanical Hyperalgesia (Randall–SelittoTest)

Mechanical hyperalgesia was assessed by evaluating the nociceptive threshold paw withdrawal following the application of mechanical pressure using an analgesiometer (IITC Life Science, Woodland Hills, CA, USA). A wedge-shaped device (area, 1.75 mm^2^) was applied to the dorsal surface of the hind paws with increasing linear pressure until the animal responded by withdrawing the paw. Three measurements were performed in the ipsilateral and contralateral paws. A cut-off threshold pressure of 250 g was programmed to prevent tissue damage. The paw withdrawal reflex was considered to represent the hypernociceptive threshold. The nociceptive paw withdrawal threshold (NPWT) was recorded in grams and defined as the percentage pressure required to provoke a withdrawal of the ipsilateral affected paw, and was calculated as follows:

(2) $$\mathrm{NPTW}\;(\%)=\frac{\mathrm{NAPWT}}{\mathrm{NAPWTW}+\mathrm{NCPWT}}\times 100$$

where NPWT was nociceptive paw withdrawal threshold, NAPWT was nociceptive affected paw–withdrawal threshold, and NCPWT was nociceptive contralateral paw withdrawal threshold [62,63].

##### Mechanical Allodynia (von Frey Test)

The Von Frey test was performed with a filament with an adapted tip, which was pressed with a constant force. For this evaluation the animals were placed in individual transparent acrylic boxes on raised platform to allow access to the lower part of their bodies. The response given at time of paw withdrawal to the filament stimulus was measured in 3 applications lasting up to 5 s each, always performed by the same evaluator [64].

### 4.4. Radiological Analysis

After euthanasia, the right hind paws of the animals were amputated and submitted to radiographs on anteroposterior and profile incidences, in order to evaluate the decrease in joint space, sclerosis of the subchondral bone and presence of osteophytes in the knees evaluated. The AP incidence was used to classify osteoarthritis by the [65], according to Table 3.

### 4.5. Histopathological Analysis of Articular Cartilage

On day 29, the knee of each animal was removed after euthanasia and fixed in 10% buffered formalin. Then, they were subjected to decalcification in 20% ethylenediaminetetraacetic acid (EDTA) for 28 days. Subsequently they were submitted to the inclusion protocol in paraffin blocks, cut into sections of 5 μm, and the proteoglycans of the organic cartilage matrix were stained specifically with 0.5% O-safranin.

The histopathological evaluation was performed according to the guidelines of the Osteoarthritis Research Society International (OARSI). The slides were analyzed blindly by two pathologists, who graded them on a scale of 0 to 6, according to the severity of the articular cartilage lesion. The classification considered the most severe lesion observed on the slide regardless of the extent of the lesion. Grade 0 indicates morphologically intact cartilage, Grade 1 indicates an intact surface with possible focal lesions or abrasion, Grade 2 shows discontinuity in the articular surface, Grade 3 shows vertical fissures, Grade 4 presents erosions, Grade 5 exhibits denudations with sclerotic bone or fibrocartilaginous tissue repair or both, and Grade 6 shows remodeling and bone deformation with changes in the contour of the articular surface [66], according to Table 4.

### 4.6. FIA-ESI-IT-MS/MSn and HPLC-ESI-IT-MS Analysis Instrumentation

For the FIA-ESI-IT-MSn assay, 10 mg of *A. chica* hydroethanolic extract was dissolved in 1 mL MeOH:H_2_O (1:1, *v*/*v*). The sample was filtered through a 0.22 μm PTFE filter, and 20 μL aliquots were injected into the LC-MS and directly into the FIA-ESI-IT-MSn system. Chromatographic profile of the crude extract of *A. chica* was performed on LCQ Fleet (Thermo Scientific^®^, San Jose, CA, USA), Kinetex^®^ C18, 100 Å (4.6 × 100 mm.; 5 μm). The mobile phase was ultra-pure water (eluent A) and acetonitrile (eluent B), both containing 0.1% formic acid in an exploratory gradient starting with 10% to 100% B in 60 min at a flow rate of 1.0 mL/min.

The sample was ionized by electrospray (ESI) and the fragments were obtained in multiple stages (MSn), in ion trap type interface. The experimental conditions were: Capillary voltage35 V, spray voltage 5000 V, capillary temperature at 350 °C, drag gas (N2), and flow 60 (arbitrary units). The acquisition range was m/z 100–2000, with two or more scanning events performed simultaneously on the spectrum. The direct flow infusion of the sample was performed on an LTQ XL Ion trap type analyzer equipped with a positive mode electrospray ionization (ESI) source (Thermo Scientific^®^, San Jose, CA, USA). A 280 °C stainless steel capillary tube, a spray voltage of 5.00 kV, a capillary voltage of 90 V, a tube lens of −100 V and a flow of 5 μL min^−1^ were used. The complete scan analysis was recorded in the m/z range of 100–1000. Multiple-stage fragmentation (ESI-MSn) was performed using the collision-induced dissociation (CID) method against helium for ion activation.

The first event was a full sweep mass spectrum to acquire data on ions in this m/z range. The second scanning event was an MS/MS experiment performed using a data-dependent scanning on the [M + H] molecules of the compounds of interest with a collision energy of 30% and an activation time of 30 ms. The product ions were then subjected to further fragmentation under the same conditions, until no further fragments were observed. The identification of the different compounds in the chromatographic profile of the hydroethanolic extract was accomplished by the mechanisms of fragmentation and comparing their mass spectral data with the literature.

### 4.7. In silico Studies

#### 4.7.1. Predictive Models and Theoretical Calculations

The metabolites identified in the *A. chica* hydroethanolic extract had their geometric, electronic, and vibrational properties optimized using the Gaussian program 09 (Gaussian, Inc., Wallingford CT) [67]. The GaussView 5.0.8 (Semichem Inc., Shawnee Mission, KS) [68] was used to obtain 3D structural models. Geometric optimization calculations were performed according to the Functional Density Theory (DFT) method, combining the functional hybrid B3LYP and the set of bases 6-31++G(d, p).

#### 4.7.2. Molecular Docking 

All docking procedures utilized the Autodock 4.2 package [69]. The structure of the cyclooxygenase 2 (COX-2) (PDB ID 1DDX) and ligands were prepared for docking simulations with AutoDock Tools version 1.5.6 [70]. Docking methodology described in literature were used [70] with some modifications [44,53]. Gasteiger partial charges were calculated after addition of all hydrogens, both ligand and macromolecule. Non-polar hydrogens from COX-2 and *A. chica* metabolites were subsequently merged. The dimensions of the cubic box in the X-, Y- and Z-axes were 120 × 120 × 120 Å, respectively, with a spacing of 0.375 Å between grid points. Grid box was centered on oxygen atom from residue Arg120 from COX-2 and the Lamarckian genetic algorithm (LGA) was chosen to search for the best conformations, with 100 runs for each compound. Initial coordinates of COX-2 and *A. chica* compounds interaction complexes were chosen based on the criterion of the better docking conformation of the cluster with the lowest energy in addition to visual inspection.

### 4.8. Statistics 

The comparison of the means of different experimental groups was performed using Student’s *t*-test or univariate analysis of variance (One-way ANOVA), followed by the Tukey test. In the evaluation of two sources of variability, bivariate variance analysis (Two-way ANOVA) was used. The *p* value <0.05 was considered as indicative of significance and the data obtained were analyzed through the software GraphPad Prism 7.0^®^ software for Windows^®^ (CA, USA). 

## 5. Conclusions

The results of the present study provide evidence that *A. chica* extract has the potential to be used in the treatment of OA. It has been shown to inhibit the enzyme COX-2; additionally, oral treatment for 25 days with *A. chica* hydroethanolic extract (50, 150, and 450 mg/kg) showed antinociceptive activity, producing improvements in incapacitation, motor activity, allodynia and hyperalgesia parameters in rats with OA experimentally induced by MIA.

The extract was able to produce radiological improvements in the affected knees; however, histopathological analysis revealed that the extract apparently did not act significantly on cartilage regeneration. Investigations into prophylactic use of the extract before OA induction may show whether the extract would be effective in preventing cartilage deterioration. Thus, more study is needed.

The results of the present study also showed that *A. chica* extract is rich in flavonoids, which have a large biological potential and may help in the treatment of OA. The in silico assays indicate a possible way of action of the compounds present in the extract, suggesting that through an interaction with cyclooxygenase-2 these compounds reduce the inflammatory process and improve OA.

## Figures and Tables

**Table 1:** HPLC-PDA-ESI-IT-MS/MS data (detected UV-vis and detected ions) and FIA-ESI-IT-MS/MS^n^ (product ions) of compounds detected in the *A. chica* leaves hydroethanolic extract.

N	RT	[M+H]	MS^n^ Fragments	Name Suggestion for Structure	Reference
**1**	4.48	299	289, 287, 160	carajurin	[17,18,19]
**2**	5.38	315	313, 289	6,7,3′-trihydroxy-5,4′-dimethoxy-flavylium	[18]
**3**	17.61	289	205, 188	2′-hydroxy-a-naphthoflavone	[20] ID BML00331
**4**	24.13	289		catechin	[21]
**5**	24.69	301	289	5,7-dimethoxy-4′-hydroxyflavone	
**6**	25.42	289		epicatechin	[21]
**7**	29.98	477	301, 289	quercetin-o-gallate	
**8**	31.12	465	301	Quercetin-o-glucoside	[22]
**9**	32.04	540	460, 301, 289	amentoflavone	[23]
**10**	34.72	479	301, 279	isorhamnetin-3-o-glucoside	[24]
**11**	36.62	301	279	chrysoeriol	[25]
**12**	37.64	287	279	kaempferol	[26]
**13**	39.65	463	330, 301	chrysoeriol-o-glucoside	[27]
**14**	41.24	317	301, 279	isorhamnetin	[24]
**15**	42.99	287	279	luteolin	[28,29]
**16**	45.25	303		6-hydroxyluteolin	[29]
**17**	46.18	477	328, 301, 279	cirsimarin	[30,31]
**18**	47.42	617	601, 301, 279	hyperin 6″ gallate	
**19**	47.64	301	279	6,7,3′,4′-tetrahydroxy-5-metoxy-flavilium	[32]
**20**	47.94	302	301, 288, 285	hispidulin	[29]
**21**	49.23			n.i.	
**22**	50.31–50.96	601		n.i.	
**23**	52.72–54.29	577	301, 289	catechin dimer	[29]
**24**	62.14	819		n.i.	
**25**	72.66	593	421, 399	feoforbide A	[33]

n.i.—not identified.

**Table 2:** Free-binding energies and inhibition constant obtained by molecular docking of the compounds identified in the *A. chica* extract with the COX-2 structure.

Ligand	ΔGbind (kcal·mol^−1^) *	Ki (μM) **
amentoflavone	−9.21	0.11
quercetin-o-gallate	−8.86	0.32
chrysoeriol-o-glucoside	−8.45	0.63
catechin dimer	−8.33	0.78
2′-hydroxy-a-naphthoflavone	−7.98	1.43
6-hydroxyluteolin	−7.95	1.48
hispidulin	−7.81	1.88
cirsimarin	−7.61	2.64
isorhamnetin-3-o-glucoside	−7.56	2.87
epicatechin	−7.49	3.25
catechin	−7.46	3.40
hyperin 6″ gallate	−7.39	3.81
6,7,3′,4′-tetrahydroxy-5-metoxy-flavylium	−7.21	5.19
6,7,3′-trihydroxy-5,4′-dimethoxy-flavylium	−7.11	6.11
carajurin	−7.06	6.71
kaempferol	−7.06	6.73
luteolin	−7.05	6.76
chrysoeriol	−6.90	8.72
quercetin-o-glucoside	−6.85	9.46
isorhamnetin	−6.72	11.86
meloxicam	−8.82	0.34

* ΔGbind: binding energy; ** Ki: inhibition constant.

**Table 3:** Classification of Kellgren–Lawrence.

**Grade 0**	**No arthrosis**—Normal radiology
**Grade I**	**Doubtful arthrosis**—Doubtful articular space narrowing and possible
**Grade II**	**Minimal osteoarthritis**—Possible joint narrowing, defined osteophytes
**Grade III**	**Moderate arthrosis**—Defined joint narrowing, multiple moderate osteophytes, some subchondral sclerosis and possible deformity in the bone contour
**Grade IV**	**Severe arthrosis**—Significant articular space narrowing, severe subchondral sclerosis, defined deformity in the bone contour and large osteophytes

**Table 4:** Osteoarthritis cartilage histopathology assessment classification system (Osteoarthritis Research Society International) [66].

Grade (Key Feature)	Subgrade (Optional)	Associated Criteria (Tissue Reaction)
Grade 0: Surface intact, Cartilage intact	No subgrade	Intact, uninvolved cartilage
Grade 1: Surface intact	1.0 Cells intact 1.5 Cell death	Matrix: superficial zone intact, edema and/or fibrillation; Cells:proliferation (clusters),hypertrophy; Reaction must be more than superficial fibrillation only.
Grade 2: Surfacediscontinuity	2.0 Fibrillation through superficial zone 2.5 Surface abrasion with matrix loss within superficial zone	As above +Discontinuity at superficial zone; ±Cationic stain matrix depletion (Safranin O or Toluidine Blue) upper 1/3 of cartilage (mid zone); ±Disorientation of chondron columns
Grade 3: Verticalfissures	3.0 Simple fissures 3.5 Branched/complex fissures	As above; ±Cationic stain depletion (Safranin O or Toluidine Blue) into lower 2/3 of cartilage (deep zone); ±New collagen formation (polarized light microscopy, Picro Sirius Red stain)
Grade 4: Erosion	4.0 Superficial zone delamination 4.5 Mid zone excavation	Cartilage matrix loss, cyst formation within cartilage matrix
Grade 5: Denudation	5.0 Bone surface intact 5.5 Reparative tissue surface present	Surface is sclerotic bone or reparative tissue including fibrocartilage.
Grade 6: Deformation	6.0 Joint margin osteophytes 6.5 Joint margin and central osteophytes	Bone remodeling; Deformation of articular surface contour (more than osteophyte formation only); Includes: microfracture and repair

## References

[1] Calixto J.B. (2005). Twenty-five years of research on medicinal plants in Latin America: A personal view. J. Ethnopharmacol..

[2] Joly C., Haddad C., Verdade L., Oliveira M., Bolzani V., Berlinck R. (2011). Diagnóstico da pesquisa em biodiversidade no Brasil. Rev. USP.

[3] Filardi F.L.R., de Barros F., Baumgratz J.F.A., Bicudo C.E.M., Cavalcanti T.B., Coelho M.A.N., Costa A.F., Costa D.P., Goldenberg R., Labiak P.H. (2018). Brazilian Flora 2020: Innovation and collaboration to meet Target 1 of the Global Strategy for Plant Conservation (GSPC). Rodriguésia.

[4] Rocha C.Q., Vilela F.C., Cavalcante G.P., Santa-Cecilia F.V., Santos-e-Silva L., dos Santos M.H., Giusti-Paiva A. (2011). Anti-inflammatory and antinociceptive effects of *Arrabidaea brachypoda* (DC.) Bureau roots. J. Ethnopharmacol..

[5] Lorenzi H., Matos F.J.A. (2008). Plantas medicinais no Brasil: Nativas e exóticas.

[6] Wang H., Wang Q., Yang M., Yang L., Wang W., Ding H., Zhang D., Xu J., Tang X., Ding H. (2018). Histomorphology and innate immunity during the progression of osteoarthritis: Does synovitis affect cartilage degradation?. J. Cell. Physiol..

[7] Ximenes A.C., Melo A.M.P., Custodio D.M.E. (2009). Dor: Princípios e Prática.

[8] Robinson W.H., Lepus C.M., Wang Q., Raghu H., Mao R., Lindstrom T.M., Sokolove J. (2016). Low-grade inflammation as a key mediator of the pathogenesis of osteoarthritis. Nat. Rev. Rheumatol..

[9] Siebuhr A.S., Bay-Jensen A.C., Jordan J.M., Kjelgaard-Petersen C.F., Christiansen C., Abramson S.B., Attur M., Berenbaum F., Kraus V., Karsdal M.A. (2016). Inflammation (or synovitis)-driven osteoarthritis: An opportunity for personalizing prognosis and treatment?. Scand. J. Rheumatol..

[10] Bjordal J.M., Klovning A., Ljunggren A.E., Slordal L. (2007). Short-term efficacy of pharmacotherapeutic interventions in osteoarthritic knee pain: A meta-analysis of randomised placebo-controlled trials. Eur. J. Pain.

[11] Tang C.H. (2019). Research of Pathogenesis and Novel Therapeutics in Arthritis. Int. J. Mol. Sci..

[12] Chou R., Helfand M., Peterson K., Dana T., Roberts C., Health O., Helfand M. (2006). Drug Class Review on Cyclo-Oxygenase (COX)-2 Inhibitors and Non-Steroidal Anti-Inflammatory Drugs (NSAIDs).

[13] Muri E., Sposito M., Metsavaht L. (2009). Antiinflamatórios não-esteroidais e sua farmacologia local. Acta Fisiátr..

[14] Sharma L., Kapoor D., Issa S. (2006). Epidemiology of osteoarthritis: An update. Curr. Opin. Rheumatol..

[15] De Rosis R.G., Kairalla M. (2010). Osteoartrite: Avaliação clínica e epidemiológica de pacientes idosos em instituição de longa permanência. Rev. Bras. Clin. Med..

[16] Kawano M.M., Araújo I.L.A., Castro M.C., Matos M.A. (2015). Assessment of quality of life in patients with knee osteoarthritis. Acta Ortop. Bras..

[17] Chapman E., Perkin A., Robinson R. (1927). The coloring matters of carajuruna. J Chem Soc.

[18] Zorn B., Garcia-Pineres A.J., Castro V., Murillo R., Mora G., Merfort I. (2001). 3-Desoxyanthocyanidins from Arrabidaea chica. Phytochemistry.

[19] Devia B., Llabres G., Wouters J., Dupont L., Escribano-Bailon M.T., de Pascual-Teresa S., Angenot L., Tits M. (2002). New 3-deoxyanthocyanidins from leaves of *Arrabidaea chica*. Phytochem. Anal..

[20] Horai H., Arita M., Kanaya S., Nihei Y., Ikeda T., Suwa K., Ojima Y., Tanaka K., Tanaka S., Aoshima K. (2010). MassBank: A public repository for sharing mass spectral data for life sciences. J. Mass Spectrom..

[21] Shen D., Wu Q., Wang M., Yang Y., Lavoie E.J., Simon J.E. (2006). Determination of the predominant catechins in *Acacia catechu* by liquid chromatography/electrospray ionization-mass spectrometry. J. Agric. Food Chem..

[22] Li Z.H., Guo H., Xu W.-B., Ge J., Li X., Alimu M., He D.J. (2016). Rapid Identification of Flavonoid Constituents Directly from PTP1B Inhibitive Extract of Raspberry (*Rubus idaeus* L.) Leaves by HPLC-ESI-QTOF-MS-MS. J. Chromatogr. Sci..

[23] Bahia M.V., David J.P., David J.M. (2010). Occurrence of biflavones in leaves of Caesalpinia pyramidalis specimens. Química Nov..

[24] Chen Y., Yu H., Wu H., Pan Y., Wang K., Jin Y., Zhang C. (2015). Characterization and Quantification by LC-MS/MS of the Chemical Components of the Heating Products of the Flavonoids Extract in Pollen Typhae for Transformation Rule Exploration. Molecules.

[25] Plazonic A., Bucar F., Males Z., Mornar A., Nigovic B., Kujundzic N. (2009). Identification and quantification of flavonoids and phenolic acids in burr parsley (*Caucalis platycarpos* L.), using high-performance liquid chromatography with diode array detection and electrospray ionization mass spectrometry. Molecules.

[26] Barbosa W.L.R., do Nascimento Pinto L., Quignard E., dos Santos Vieira J.M., Silva J.O.C., Albuquerque S. (2008). *Arrabidaea chica* (HBK) Verlot: Phytochemical approach, antifungal and trypanocidal activities. Rev. Bras. Farmacogn..

[27] Iwashina T., Smirnov S.V., Damdinsuren O., Kondo K. (2010). *Saussurea* Species from the Altai Mountains and Adjacent Area, and Their Flavonoid Diversity. Bull. Natl. Mus. Nat. Sci. Ser. B.

[28] Takemura O.S., Iinuma M., Tosa H., Miguel O.G., Moreira E.A., Nozawa Y. (1995). A flavone from leaves of *Arrabidaea chica* f. cuprea. Phytochemistry.

[29] Siraichi J.T.G., Felipe D.F., Brambilla L.Z.S., Gatto M.J., Terra V.A., Cecchini A.L., Cortez L.E.R., Rodrigues-Filho E., Cortez D.A.G. (2013). Antioxidant capacity of the leaf extract obtained from *Arrabidaea chica* cultivated in Southern Brazil. PLoS ONE.

[30] Hasrat J.A., Pieters L., Claeys M., Vlietinck A., De Backer J.P., Vauquelin G. (1997). Adenosine-1 active ligands: Cirsimarin, a flavone glycoside from *Microtea debilis*. J. Nat. Prod..

[31] Lee J., Rodriguez J.P., Lee K.H., Park J.Y., Kang K.S., Hahm D.-H., Huh C.K., Lee S.C., Lee S. (2017). Determination of flavonoids from *Cirsium japonicum* var. maackii and their inhibitory activities against aldose reductase. Appl. Biol. Chem..

[32] Lima J.C.S., de Oliveira R.G., Silva V.C., de Sousa P.T.J., Violante I.M.P., Macho A., de Oliveira Martins D.T. Anti-inflammatory activity of 4′,6,7-trihydroxy-5-methoxyflavone from *Fridericia chica* (Bonpl.) L.G.Lohmann. Nat. Prod. Res..

[33] Miranda N., Gerola A.P., Novello C.R., Ueda-Nakamura T., de Oliveira Silva S., Dias-Filho B.P., Hioka N., de Mello J.C.P., Nakamura C.V. (2017). Pheophorbide a, a compound isolated from the leaves of *Arrabidaea chica*, induces photodynamic inactivation of *Trypanosoma cruzi*. Photodiagnosis Photodyn. Ther..

[34] Liu X., Machado G.C., Eyles J.P., Ravi V., Hunter D.J. (2018). Dietary supplements for treating osteoarthritis: A systematic review and meta-analysis. Br. J. Sports Med..

[35] Maksimović Z., Samardžić S. (2018). Herbal Medicinal Products in the Treatment of Osteoarthritis. Osteoarthritis Biomarkers and Treatments.

[36] Ribeiro A.F.C. (2012). Avaliação das atividades anti-inflamatoria, antiangiogenica e anti-tumoral de extratos de *Arrabidaea chica* (Humb. & Bonpl.) B. Verlot.. Ph.D. Thesis.

[37] Michel A.F.R.M., Melo M.M., Campos P.P., Oliveira M.S., Oliveira F.A.S., Cassali G.D., Ferraz V.P., Cota B.B., Andrade S.P., Souza-Fagundes E.M. (2015). Evaluation of anti-inflammatory, antiangiogenic and antiproliferative activities of Arrabidaea chica crude extracts. J. Ethnopharmacol..

[38] Lima F.C.V.M. (2018). Efeito de Arrabidaea chica Verlot na dor neuropática póstraumática em ratos.. Ph.D. Thesis.

[39] Hohjoh H., Inazumi T., Tsuchiya S., Sugimoto Y. (2014). Prostanoid receptors and acute inflammation in skin. Biochimie..

[40] Choudhary D., Kothari P., Tripathi A.K., Singh S., Adhikary S., Ahmad N., Kumar S., Dev K., Mishra V.K., Shukla S. (2018). Spinacia oleracea extract attenuates disease progression and sub-chondral bone changes in monosodium iodoacetate-induced osteoarthritis in rats. BMC Complement. Altern. Med..

[41] Chien T.-Y., Huang S.K.-H., Lee C.-J., Tsai P.-W., Wang C.-C. (2016). Antinociceptive and Anti-Inflammatory Effects of Zerumbone against Mono-Iodoacetate-Induced Arthritis. Int. J. Mol. Sci..

[42] Verri W.A.J., Cunha T.M., Parada C.A., Poole S., Cunha F.Q., Ferreira S.H. (2006). Hypernociceptive role of cytokines and chemokines: Targets for analgesic drug development?. Pharmacol. Ther..

[43] Kumari R.R., More A.S., Gupta G., Lingaraju M.C., Balaganur V., Kumar P., Kumar D., Sharma A.K., Mishra S.K., Tandan S.K. (2015). Effect of alcoholic extract of *Entada pursaetha* DC on monosodium iodoacetate-induced osteoarthritis pain in rats. Indian J. Med. Res..

[44] Calado G.P., Lopes A.J.O., Costa Junior L.M., Lima F.D.C.A., Silva L.A., Pereira W.S., do Amaral F.M.M., Garcia J.B.S., Cartágenes M.D.S.D.S., Nascimento F.R.F. (2015). *Chenopodium ambrosioides* L. Reduces Synovial Inflammation and Pain in Experimental Osteoarthritis. PLoS ONE.

[45] Seeram N.P., Momin R.A., Nair M.G., Bourquin L.D. (2001). Cyclooxygenase inhibitory and antioxidant cyanidin glycosides in cherries and berries. Phytomedicine.

[46] Dougados M., Nguyen M., Berdah L., Mazieres B., Vignon E., Lequesne M. (2001). Evaluation of the structure-modifying effects of diacerein in hip osteoarthritis: ECHODIAH, a three-year, placebo-controlled trial. Evaluation of the Chondromodulating Effect of Diacerein in OA of the Hip. Arthritis Rheum..

[47] Dieppe P.A., Lohmander L.S. (2005). Pathogenesis and management of pain in osteoarthritis. Lancet.

[48] Manach C., Scalbert A., Morand C., Rémésy C., Jiménez L. (2004). Polyphenols: Food sources and bioavailability. Am. J. Clin. Nutr..

[49] Marques J.I., Alves J.S.F., Torres-Rêgo M., Furtado A.A., da Silva Siqueira E.M., Galinari E., de Souza Araújo D.F., Guerra G.C.B., de Azevedo E.P., Fernandes-Pedrosa M.D.F. (2018). Phytochemical Analysis by HPLC–HRESI-MS and Anti-Inflammatory Activity of Tabernaemontana catharinensis. Int. J. Mol. Sci..

[50] Lopes A.J.O., Vasconcelos C.C., Pereira F.A.N., Silva R.H.M., Queiroz P.F.S., Fernandes C.V., Garcia J.B.S., Ramos R.M., Rocha C.Q., Lima S.T.J.R.M. (2019). Anti-Inflammatory and Antinociceptive Activity of Pollen Extract Collected by Stingless Bee *Melipona fasciculata*. Int. J. Mol. Sci..

[51] Rowlinson S.W., Kiefer J.R., Prusakiewicz J.J., Pawlitz J.L., Kozak K.R., Kalgutkar A.S., Stallings W.C., Kurumbail R.G., Marnett L.J. (2003). A novel mechanism of cyclooxygenase-2 inhibition involving interactions with Ser-530 and Tyr-385. J. Biol. Chem..

[52] Xu S., Hermanson D.J., Banerjee S., Ghebreselasie K., Clayton G.M., Garavito R.M., Marnett L.J. (2014). Oxicams bind in a novel mode to the cyclooxygenase active site via a two-water-mediated H-bonding Network. J. Biol. Chem..

[53] Silva R.H.M., Lima N.D.F.M., Lopes A.J.O., Vasconcelos C.C., de Mesquita J.W.C., de Mesquita L.S.S., Lima F.C.V.M., Ribeiro M.N.D.S., Ramos R.M., Cartágenes M.D.S.D.S. (2017). Antinociceptive activity of *Borreria verticillata:* In vivo and in silico studies. Front. Pharmacol..

[54] Oh J., Rho H.S., Yang Y., Yoon J.Y., Lee J., Hong Y.D., Kim H.C., Choi S.S., Kim T.W., Shin S.S. (2013). Extracellular signal-regulated kinase is a direct target of the anti-inflammatory compound amentoflavone derived from *Torreya nucifera*. Mediators Inflamm..

[55] Banerjee T., Van der Vliet A., Ziboh V.A. (2002). Downregulation of COX-2 and iNOS by amentoflavone and quercetin in A549 human lung adenocarcinoma cell line. Prostaglandins. Leukot. Essent. Fatty Acids.

[56] Sakthivel K.M., Guruvayoorappan C. (2013). Amentoflavone inhibits iNOS, COX-2 expression and modulates cytokine profile, NF-kappaB signal transduction pathways in rats with ulcerative colitis. Int. Immunopharmacol..

[57] Nagy E., Vajda E., Vari C., Sipka S., Farr A.-M., Horvath E. (2017). Meloxicam ameliorates the cartilage and subchondral bone deterioration in monoiodoacetate-induced rat osteoarthritis. PeerJ.

[58] Fernihough J., Gentry C., Malcangio M., Fox A., Rediske J., Pellas T., Kidd B., Bevan S., Winter J. (2004). Pain related behaviour in two models of osteoarthritis in the rat knee. Pain.

[59] Silva A., Andersen M.L., Tufik S. (2008). Sleep pattern in an experimental model of osteoarthritis. Pain.

[60] Monville C., Torres E.M., Dunnett S.B. (2006). Comparison of incremental and accelerating protocols of the rotarod test for the assessment of motor deficits in the 6-OHDA model. J. Neurosci. Methods.

[61] Schott E., Berge O.G., Angeby-Moller K., Hammarstrom G., Dalsgaard C.J., Brodin E. (1994). Weight bearing as an objective measure of arthritic pain in the rat. J. Pharmacol. Toxicol. Methods.

[62] Santos-Nogueira E., Redondo Castro E., Mancuso R., Navarro X. (2012). Randall-Selitto test: A new approach for the detection of neuropathic pain after spinal cord injury. J. Neurotrauma.

[63] Randall L.O., Selitto J.J. (1957). A method for measurement of analgesic activity on inflamed tissue. Arch. Int. Pharmacodyn. Ther..

[64] Moller K.A., Johansson B., Berge O.G. (1998). Assessing mechanical allodynia in the rat paw with a new electronic algometer. J. Neurosci. Methods.

[65] Kellgren J.H., Lawrence J.S. (1957). Radiological assessment of osteo-arthrosis. Ann. Rheum. Dis..

[66] Pritzker K.P.H., Gay S., Jimenez S.A., Ostergaard K., Pelletier J.-P., Revell P.A., Salter D., van den Berg W.B. (2006). Osteoarthritis cartilage histopathology: Grading and staging. Osteoarthr. Cartil..

[67] Frisch M.J., Trucks G.W., Schlegel H.B., Scuseria G.E., Robb M.A., Cheeseman J.R., Scalmani G., Barone V., Petersson G.A., Nakatsuji H. (2009). Gaussian 09.

[68] Dennington R., Keith T.A., Millam J.M. (2016). GaussView5.

[69] Morris G.M., Goodsell D.S., Halliday R.S., Huey R., Hart W.E., Belew R.K., Olson A.J. (1998). Automated docking using a Lamarckian genetic algorithm and an empirical binding free energy function. J. Comput. Chem..

[70] Morris G.M., Huey R., Olson A.J. (2008). Using AutoDock for ligand-receptor docking. Curr. Protoc. Bioinform..

